# Computational Studies Applied to Flavonoids against Alzheimer's and Parkinson's Diseases

**DOI:** 10.1155/2018/7912765

**Published:** 2018-12-30

**Authors:** Alex France M. Monteiro, Jéssika De O. Viana, Anuraj Nayarisseri, Ernestine N. Zondegoumba, Francisco Jaime B. Mendonça Junior, Marcus Tullius Scotti, Luciana Scotti

**Affiliations:** ^1^Postgraduate Program in Natural and Synthetic Bioactive Products, Federal University of Paraíba, João Pessoa, PB, Brazil; ^2^In Silico Research Laboratory, Eminent Bioscience, Inodre - 452010, Madhya Pradesh, India; ^3^Bioinformatics Research Laboratory, LeGene Biosciences, Indore - 452010, Madhya Pradesh, India; ^4^Department of Organic Chemistry, Faculty of Science, University of Yaounde I, PO Box 812, Yaoundé, Cameroon; ^5^Laboratory of Synthesis and Drug Delivery, Department of Biological Science, State University of Paraiba, João Pessoa, PB, Brazil; ^6^Teaching and Research Management-University Hospital, Federal University of Paraíba, João Pessoa, PB, Brazil

## Abstract

Neurodegenerative diseases, such as Parkinson's and Alzheimer's, are understood as occurring through genetic, cellular, and multifactor pathophysiological mechanisms. Several natural products such as flavonoids have been reported in the literature for having the capacity to cross the blood-brain barrier and slow the progression of such diseases. The present article reports on *in silico* enzymatic target studies and natural products as inhibitors for the treatment of Parkinson's and Alzheimer's diseases. In this study we evaluated 39 flavonoids using prediction of molecular properties and *in silico* docking studies, while comparing against 7 standard reference compounds: 4 for Parkinson's and 3 for Alzheimer's. Osiris analysis revealed that most of the flavonoids presented no toxicity and good absorption parameters. The Parkinson's docking results using selected flavonoids as compared to the standards with four proteins revealed similar binding energies, indicating that the compounds 8-prenylnaringenin, europinidin, epicatechin gallate, homoeriodictyol, capensinidin, and rosinidin are potential leads with the necessary pharmacological and structural properties to be drug candidates. The Alzheimer's docking results suggested that seven of the 39 flavonoids studied, being those with the best molecular docking results, presenting no toxicity risks, and having good absorption rates (8-prenylnaringenin, europinidin, epicatechin gallate, homoeriodictyol, aspalathin, butin, and norartocarpetin) for the targets analyzed, are the flavonoids which possess the most adequate pharmacological profiles.

## 1. Introduction

Neurodegenerative diseases (NDDs) arise as a progressive loss of neuron structure and function, resulting in muscle weakness and deterioration of the body's physiological functions [1, 2]. During this process, postmitotic cells undergo cell death, leading to cellular apoptosis signaling and further oxidative stress [3]. In addition to neuronal loss, other pathological genetic, biochemical, and molecular factors affect the progression of the disease. Recent studies have demonstrated the presence of proteins in the brains of the affected (involved in the process of NDDs), with modified physicochemical properties [4]. NDDs include Alzheimer's disease (AD), Parkinson's disease (PD), Huntington disease (HD), schizophrenia, amyotrophic lateral sclerosis (ALS), seizure disorders, and head injuries along with other systemic disorders [5].

Phytochemicals are a diversified group of naturally occurring bioactive compounds in plants; they include flavonoids, alkaloids, terpenoids, lignans, and phenols. Since they have a wide range of chemical, biochemical, and molecular characteristics, phytochemicals are of considerable interest for treating NDDs. Phytochemicals are promising candidates for various pathological conditions involving modulation of multiple signal pathways and serving as antioxidant and anti-inflammatory agents [6], agents against cancer and neurodegenerative diseases [7–9], or as antifungal agents [10]. Several studies have addressed the protective activity of natural derivatives such as alkaloids when applied to neurodegenerative diseases such as Alzheimer's and Parkinson's [11]; genistein brings neuroprotective effects [12, 13]; hesperetin presents potent antioxidant and neuroprotective effects [14]; quercetin [15] and xanthones present multifunctional activities against Alzheimer's disease [16].

Flavonoids fit the NDDs profile, and in a process dependent on the suppression of lipid peroxidation, inhibition of inflammatory mediators, modulation of gene expression, and activation of antioxidant enzymes, flavonoids help maintain the endogenous antioxidant status of neurons, protecting them from neurodegeneration [17, 18]. Based on their chemical structure, they are classified into several categories including flavanols, flavonols, flavones, flavanones, isoflavones, anthocyanidins, and chalcones [19].

This article focuses on flavonoids found in the literature for anti-Parkinson and anti-Alzheimer activity, including targets involved in the degenerative process of each disease. Molecular docking studies detail the structural parameters involved that best contribute to the activity of such compounds. This study facilitates knowledge as applied to two NDDs concerning flavonoid structural enhancements and the pharmacophores involved in the receptor-protein complex.

## 2. Parkinson's Disease

Parkinson's disease (PD) is the second most common neurodegenerative disease globally and has been increasing considerably without evidence of cure [20, 21]. PD is reported as a loss of dopaminergic neurons located in the substantia nigra (SN) and affects 1-2% of people over the age of 60 [22]. Estimates of the disease range from 5 to 35 new cases per 100,000 individuals [23]; this increases with age [24]. The prevalence of PD is increasing considerably, corroborating a doubling by the year 2030 [25].

To characterize PD, progressive degeneration of dopaminergic (DA) neurons causing depletion of striatal dopamine and formation of Lewy bodies in the substantia nigra (SN) are the principal neuropathological correlations of motor damage in PD. The symptoms include resting tremor, rigidity, bradykinesia, gait difficulty, postural instability, and behavioral problems [26]; nonmotor symptoms include depression, anxiety, emotional changes, cognitive impairment, sleep difficulty, and olfactory dysfunction [27]. There are several studies that report neurodegenerative factors such as neuroinflammation [28] and cytotoxic factors such as IL1, NO, ROS, and TNF [29].

The treatment of PD focuses on carbidopa to replace dopamine, levodopa drugs, monoamine oxidase B inhibitors, dopamine agonists, catechol-o-methyltransferase inhibitors, anticholinergics, and amantadine [30]. Levodopa is the single most used drug to treat Parkinson's disease [31]. However, these drugs cause many side effects [32], and they usually lead to other complications, yet without curing or stopping disease progression. The search for new therapeutic agents with few side effects is essential.

The use of natural products against PD has intensified in recent years, chiefly compounds derived from plants, since they are known to have fewer side effects than synthetic compounds [33, 34]. These advances in the treatment of PD give the disease a chance to be administered effectively, leading to symptom control and improvement of patient quality of life, often for decades after onset of the disease.

### 2.1. Molecular Docking Applied to Natural Products for Parkinson's Disease

Molecular docking studies are based on joining a particular ligand to a receptor region, providing information about conformation, orientation, and organization at the receptor site [35]. Studies using computational chemistry to predict potential inhibitors for neurodegenerative diseases have been reported in the literature [36–38], and studies involving molecular docking have been reported in the literature for Parkinson's disease and flavonoid derivatives [39].

Desideri et al. [40] reported the *in vitro* and *in silico* activity of a series of homo-isoflavonoids as potent inhibitors of human monoamine oxidase-B. Presenting better *in vitro* results than the standard drug, selegiline, (E)-3-(4-(Dimethylamino)benzylidene)chroman-4-one and (E)-5,7-dihydroxy-3-(4-hydroxybenzylidene)chroman-4-one also demonstrated selectivity and high potency during the *in silico* studies, interacting with hydrogen and hydrophobic bonds at the active site.

Our research group applied ligand-based-virtual screening together with structure based-virtual screening (docking) for 469 alkaloids of the Apocynaceae family in a study of human AChE inhibitory activity [41]. As a result, 9 alkaloids presenting better inhibition profiles for both Parkinson's and Alzheimer's (dihydro-cylindrocarpine, 14,19-dihydro-11-methoxycondylocarpine, Di (demethoxycarbonyl) tetrahydrosecamine, tetrahydrosecamine, 16-demethoxycarbonyltetrahydrosecamine, 16-hydroxytetrahydrosecamine, usambarensine, 4′,5′,6′,17-tetrahydro-usambarensine-N-oxide, and 6,7-seco-angustilobine) were selected for future studies.

Baul and Rajiniraja [42] performed a molecular docking study using flavonoids such as quercetin, epigallocatechin gallate (EGCG), and acacetin to predict inhibitory activities and their ability to inhibit the enzyme *α*-synuclein. The results showed that the flavonoids present low energy value interactions with residues Lys45, Lys43, Lys32, and Val40, being essential for activity in this protein.

*In silico* studies involving Parkinson's disease anti-inflammatory activity have also been targeted for novel bioactive compounds. As a general rule for anti-inflammatory activity, both hydrogen and *π*-*π* hydrophobic interactions between the active site of the macromolecule and the compounds are essential. Madeswaran et al. [43] reported the inhibition activity of nine flavonoids (morin, naringenin, taxifolin, esculatin, daidzein, genistein, scopoletin, galangin, and silbinin) against human lipoxygenase enzyme. The flavonoid interactions especially those of morin were similar to Azelastine, a flavonoid already reported in the literature for lipoxygenase inhibition activity, thus defining amino acids Tyr359, Gln358, and Gln539 as critical to the activity of these compounds.

### 2.2. Targets in Parkinson's Disease

#### 2.2.1. Adenosine A_2A_ Receptors

Adenosine receptors are members of the G protein-coupled receptor superfamily and considered potential targets for treatment of numerous diseases. Adenosine binds four types of G-protein receptors known as A_1_, A_2A_, A_2B_, and A_3_ all with distribution in the brain. A_2A_ has a more specific and abundant distribution in the basal ganglia. This selective distribution for receptors can help guarantee fewer adverse effects and make nondopaminergic antagonists more promising for the treatment of PD [44].

The A_2A_ adenosine receptor (A_2A_AR) is highly expressed in the basal ganglia and depends on Gs and other protein interactions for signal interpretation [45]. In mammals, high expression of this protein is found in the striatum in the basal ganglia, with an important route for the regulation of dopaminergic transmission [46]. The A_2A_ receptor subtype presents signaling involving activation of serine/threonine kinase [47, 48], which modulates phosphorylation of ionotropic glutamate receptors [49, 50]. The A_2A_ receptor may provide improvement in motor abnormalities for patients with PD, by controlling hyperphosphorylation of the glutamatergic receptor.

Indeed, five A_2A_ receptor antagonists are now in clinical trials (phases I to III) for Parkinson's disease, and other antagonists have been reported in the literature [51]. The use of these receptors is due to various preclinical studies which have shown that adenosinergic neuromodulation antagonizes dopaminergic neurotransmission in aspects relevant to motor control. The adenosine A_2A_ receptor activates adenylyl cyclase and certain voltage-sensitive Ca^2+^ channels [52]. These receptors are expressed in the GABAergic neurons and in glutamatergic neuronal terminals [53].

Schwarzschild et al. [54] proposed an anti-Parkinson activity reactive mechanism for the A_2A_ receptor. In the normal state, the dopamine of the neurons is found in the substantia nigra and acts on two receptors: D1 receptors (direct stimulatory pathway) and D2 receptors (indirect inhibitory pathway). Adenosine, which is released by A_2A_ receptors, stimulates neurons at the D2 receptor pathway. In degenerative processes, as is the case in PD, the central nervous system (CNS) degeneration blocks the entry of striatum dopamine, which increases GABA's inhibitory influence, consequently mitigating PD motor deficits.

The restriction of striatum region expression contributes to fewer side effects in PD patients [55–57]. Several studies have reported the activity of nondopaminergic A_2A_ receptor antagonists [58, 59], a good target for the development of anti-Parkinson drugs.

#### 2.2.2. *α*-Synuclein

A 140 amino acid protein, *α*-synuclein is commonly located in presynaptic terminals [60, 61]. Alpha-synuclein represents the most abundant protein in Lewy bodies (LB), cytoplasmic inclusions found in PD and in LB dementia (LBD), which have a little understood physiology. The synuclein family has three members, *α*-synuclein, *β*-synuclein, and *γ*-synuclein, ranging from 127 to 140 amino acids, with about 55 to 62% of homologous sequences, and where *α* and *β* have an identical carboxy-terminal domain. These proteins are commonly found in nerve terminals, close to synaptic vesicles; *β*-synucleins are present in almost all nerve cells [62].

Among the factors that influence *α*-synuclein abnormalities, genetic factors (protein gene, PARK3, and PARK4 locus mutations) and environmental factors (oxidative damages) often lead to errors in the ordering and conformation of *α*-synuclein filaments [63].

Recent studies report a mutation of alanine to threonine at position 53 of the protein gene causing a rare and familial form of PD in four families [64]. The identification of this mutation in autosomal dominant families of inherited Parkinson's led to the discovery of a new target for PD pathology.

Olanow and Brundin [65] provided evidence of *α*-synuclein activity in prion-like proteins acting in PD, thus suggesting new studies for the development of inhibitors. Recent studies have reported that a doubling or tripling of the *α*-synuclein gene leads to a similar type of PD [66, 67]. Mutagenic studies involved in the *α*-synuclein response associate and reinforce the hypothesis that mutations are involved in the pathogenesis of PD.

#### 2.2.3. Catechol-O-Methyltransferase

The enzyme catechol-O-methyltransferase, also known as COMT, is as an important enzyme involved in biochemistry, pharmacology, and genetic mechanisms. Methylation of endogenous catecholamines, as well as other catechols, is catalyzed by the enzyme catechol-O-methyltransferase (COMT). COMT transfers the methyl group of S-adenosylmethionine (SAM) to the *meta-* or *para*-hydroxyl group present in catechols [68, 69]; COMT is considered a SAM-dependent methyltransferase [70]. COMT substrates involve both endogenous and exogenous catechols, such as dopamine, norepinephrine, and epinephrine. In the brain, COMT is involved in mental processes, as studies have reported for Parkinson's disease [71]. COMT is considered a target for study and development of new anti-Parkinson drugs using coadministration with levodopa [72, 73]. The enzyme has two forms: a soluble form, known as S-COMT, presenting 221 residues; and a second form, known as membrane based (MB-COMT), exhibiting 50 residues at the N-terminus [74]. The COMT active site has a SAM binding site and an S-COMT catalytic site. In addition, the presence of Mg^2+^ in the catalytic site is responsible for converting catechol hydroxyl groups to substrates [68].

The COMT enzyme has the single domain structure containing *α* and *β* moieties, where 8 helices are disposed around a central *β* sheet. The active site of the enzyme is composed of an S-adenosyl-L-methionine-(AdoMet-) binding domain, similar to a Rossmann fold, and present in numerous proteins that interact with nucleotides [68].

The catechol-O-methyltransferase (COMT) gene encodes an enzyme that performs catecholamine (such as dopamine, epinephrine, and norepinephrine) degradation [75]; this process is depressed in patients with PD. The COMT gene is located on chromosome 22q11, which has been reported as one of the major loci related to schizophrenia [76]. Recent studies have shown a polymorphism at codon 158 (Val158Met, called rs4680) that influences the COMT enzyme, by decreasing its activity [77], and which interferes with executive cognitive performance [78, 79].

#### 2.2.4. Monoamine Oxidase B

The enzyme monoamine oxidase B (MAO-B) has been reported as a therapeutic target for the treatment of Parkinson's disease [80, 81] and is also a brain glial biomarker [82]. Studies have shown that MAO is located in the outer mitochondrial membrane, in the liver, and in the brain [83] and presents FAD as a cofactor in its active site, where irreversible MAO inhibitors bind, such as rasagiline.

MAO's mechanism of reaction involves oxidative deamination of primary, secondary, and tertiary amines, to the corresponding aldehyde, and free amine with the generation of hydrogen peroxide. As for the aldehyde, this is metabolized by the enzyme aldehydedehydrogenase, producing acids such as 5-hydroxyindole acetic acid (5-HIAA) or dihydroxy-phenyl-acetic acid (DOPAC), metabolites used as MAO activity drugs. MAO also produces hydrogen peroxide, leading to oxidative stress and neuronal cell death [84, 85].

MAO can be found in two isoforms, known as isoform A and isoform B, with differences that are of great pharmacological importance [86]. Isoform A is located next to catecholaminergic neurons, whereas the B isoform is located in neurotransmitters. Among the two subtypes, MAO-B is one of the enzymes that oxidize the neurotransmitter dopamine in addition to metabolizing other amines. This enzyme is found in large numbers in astrocytes but is also present in serotonin neuron cell bodies, whereas MAO-A is located in neurons in the brain [87]. Isoform A is inhibited by low concentrations of clorgiline, while MAO-B is inhibited by selegiline and rasagiline [88–90], drugs used to elevate brain dopamine by inhibiting its breakdown and promoting beneficial symptomatic effects for the patient.

Studies have reported the expression of MAO-B in human brains or more precisely in the substantia nigra of patients affected by PD [91, 92]. Human MAO-B presents two cavities in its structure, and the FAD coenzyme is present in the active site. The N5 atom is present in the external region, and the residues Tyr398 and Tyr435 play important roles in hMAO-B catalytic activity [93]. The inhibition of MAO-B using rasagiline may promote increased dopaminergic activity of the striatum, leading to symptomatic benefits due to interference in dopamine degradation. Improvements also result from decreased free radicals as generated from dopamine oxidation. The development of selective and reversible MAO-B inhibitors may reduce undesirable adverse effects and present long-term efficacy in neurodegenerative disease treatment.

## 3. Alzheimer's Disease

Alzheimer's disease (AD) is a progressive neurodegenerative disease common in older people (from 60 years of age and upwards). It consists in memory loss and gradual impairment of cognitive function due to mainly cholinergic neuron death, which makes accomplishment of daily activities difficult, leading the patient to dependence for the basic activities of their daily routine. Because the neurological impairment compromises the autonomic nervous system (ANS), it eventually leads to death. [94–98].

One of the symptoms of AD is dementia, and according to the World Health Organization (WHO) Bulletin, AD is the main pathology responsible for up to 70% of individuals with dementia. WHO estimates that more than 47 million people suffer from dementia, and more than half are from underdeveloped countries. Alzheimer's has no cure and its treatment consists of trying to slow the progression of the disease and offer symptomatic relief [99, 100].

Alzheimer's is clinically explained by neuronal decreases linked to deficient synthesis of acetylcholine (ACh) involved in memory, learning, and SNA. Thus, studies commonly aim at inhibiting acetylcholinesterase (AChE) to prevent ACh breakdown and consequent loss of memory and cognitive functions [101–104].

### 3.1. Molecular Docking Applied to Natural Products for Alzheimer's Disease

Bioactive beta-secretase-1 (BACE1) inhibitors are currently being studied as therapeutic targets. BACE1 inhibition prevents the amyloid *β*-amyloid peptide (A*β*) from increasing, preventing cleavage of localized amyloid precursor protein (APP), and thus portion C99 enters the membrane while the (sAPP*β*) portion enters the extracellular environment. Inhibition of BACE1 is a therapeutic alternative that inhibits the evolution of AD. This hypothesis has been known since the 1990s as “amyloid cascade” because it consists of a set of neuropathological events that occur in chain, initiated by the accumulation of A*β*, followed by the dysfunction of Tau proteins (which normally stabilize neuronal microtubules), which results in cell death through the agglomeration of Tau proteins in the cell; this compromises both dendrite and the neuronal cell body functions [105–109].

In a molecular docking study [110] to identify molecules that potentiate Alzheimer's inhibition in the BACE1 target, docking of 14 molecules using Molex Virtual Docker was performed with PDB ID 2XFJ and presented interactions with amino acid residues Thr292, Asp93, Asp289, Thr293, Gln134, Asn294, and Thr133. For the compounds studied, hydrogen bonds and hydrophobic interactions with these residues favored inhibitory activity.

**Figure 1:**
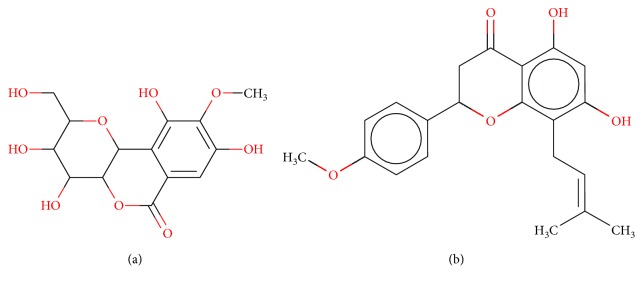
2D structure of Alzheimer's disease inhibitors. (a) Bergenin. (b) 5,7-dihydroxy-4′-methoxy-8-prenylflavanone.

Barai et al. [111] using the GOLD suite v.5 program analyzed molecular docking interactions of bergenin (Figure 1(a)) 2 with the objective of highlighting its neuroprotective effects against AD. The docking data in this study were obtained from interactions of the natural product with acetylcholinesterase (PDB ID 1B41), butyrylcholinesterase (PDB ID 1P0I), Tau protein kinase 1 (PDB ID 1J1B), and BACE-1 (PDB ID 1FKN). The docking results were compared with the standard drugs donepezil, galantamine, and physostigmine. In the AChE target interactions, hydrogen bonds were present with residues Val340, Gly342, and Phe346; for the BuChE target, hydrogen bond interactions appeared with residues Asn245, Phe278, Val280, and Pro281; for GSK-3*β*, hydrogen bond interactions appeared with residues Ile62, Gly68, Lys85, Leu132, Asp133, Tyr134, Val135, Arg141, and Asp200, with hydrogen bonds also appearing in most of the residues; and finally for BACE1, hydrogen bond interactions with the amino acids Asp32, Gly34, Asp228, Thr231, and Arg235 were present. In each target, bergenin presented amino acid residue interactions similar to those of the standard drugs studied: Arg24, Lys32, Val340, Gly342, Ala343, and Phe346.

Das et al. [112] performed *in silico* molecular docking studies with 5,7-dihydroxy-4′-methoxy-8-prenylflavanone (Figure 1(b)) using the FlexX of Biosolveit LeadIT program along with the drugs donepezil, galantamine, rivastigmine, tacrine, huperzine, methoxytaxine, and others. The target (PDB ID 5HF6) was chosen with the help of the PharmMapper tool (http://lilab.ecust.edu.cn) and is involved in inhibition of acetylcholinesterase. The aim of this study was to predict anti-Alzheimer activity through molecular docking and QSAR. As a conclusion of this research, the studied flavonoid presented a better ligand-receptor score (−13.576 kJ.mol^−1^) than 9 of the 21 controls used for comparison.

### 3.2. Targets in Alzheimer's Disease

#### 3.2.1. Glycogen Synthase Kinase 3 (GSK3)

Glycogen synthase kinase-3 (GSK-3) is a protein responsible for the addition of phosphate molecules to serine and threonine residues [113–115] and is generally encoded by two GSK3*α* and GSK3*β* genes. GSK3*β* phosphorylates the Tau protein and its expression is related to diseases such as Alzheimer's, cancer, and diabetes [113–116].

GSK3*β* phosphorylates the Tau protein; amino acid residue Tyr216 activates protein kinase, while Ser9 contributes to inhibition. Studies by Nicolia et al. [117] in neuroblastoma cells, analyzing hypomethylation in postmortem frontal cortex, showed that patients with initial AD present inactive GSK3*β* decreases, whereas patients in the pathological stage V-VI level present large increases in inactive GSK3*β*.

According to Chinchalongporn et al. [118] who analyzed the inhibitory effect of melatonin on the production of *β*-amyloid peptide, activation of the GSK3*β* gene contributes to the formation of A*β* and neuritic plaque and thus a large increase in Tau phosphorylation.

#### 3.2.2. TNF-*α* Converting Enzyme (TACE)

Two factors are associated with the incidence of Alzheimer's, the increase of *β*-amyloid plaques that form and impede neurotransmissions and the presence of neurofibrillary structures containing Tau in the brain. Tumor necrosis factor-*α* (TNF-*α*) is a transmembrane protein that when undergoing TACE (TNF-*α* converting enzyme) action releases its extracellular domain or soluble TNF-*α*. TNF*sα* is a signaling protein; its deregulation is directly related to neuronal degeneration and inflammation [119, 120]. Many studies show that neuroinflammation can trigger pathological processes, including AD. TNF-*α* is usually maintained at very low concentrations, but with the development of AD the levels increase. [120–123].

#### 3.2.3. Human Angiotensin-Converting Enzyme (ACE)

ACE is a zinc metalloenzyme that helps regulate blood pressure and body fluids, by converting the hormone angiotensin I into angiotensin II, a potent vasoconstrictor which is widely used in cardiovascular disease therapies such as degradation of *β*-amyloid [124–126]. ACE is a peptide and widely distributed as an ectoenzyme in vascular endothelial cell membranes, in epithelial and neuroepithelial cells, and also in its plasma soluble form. Studies have shown that ACE inhibition is a promising therapeutic target for Alzheimer's because angiotensin II in some studies has blocked memory consolidation [127–130].

#### 3.2.4. BACE1 Inhibitor

BACE1, a *β*-secretase involved in the production of *β*-amyloid peptide, is the cleavage enzyme of the amyloid precursor protein site 1 and is very important in AD studies. BACE1 has become an increasingly well-studied pharmacological target; many research groups seek bioactives with inhibitory action against this enzyme, yet major problems with inhibitory drugs that cross the blood-brain barrier remain [131–134]. Studies with mice show that BACE1 inhibitors are efficient in combating new A*β* plaques but inefficient against growth of existing plaques, suggesting early treatment with the aim of preventing initial plaque formation [135, 136].

## 4. Materials and Methods

### 4.1. Data Set

From the literature, we selected the set of 39 flavonoid structure, known for their antioxidant action. The compounds were submitted to molecular modeling and molecular docking tools to provide their important structural information and activity as multitarget compounds. Data for the physicochemical characteristics of the compounds has been reported (Table 1).

### 4.2. Molecular Modeling

All of the structures were drawn in HyperChem for Windows v. 8.0.5 (HyperChem, 2009) [137], and their molecular geometries were minimized using the molecular mechanics MM^+^ force field, without restrictions for aromatic form conversions, and clean molecular graphing in three dimensions. The optimized structures were subjected to conformational analysis using a random search method with 1000 interactions, 100 cycles of optimization, and the 10 lowest minimum energy conformers. The compounds were saved in the MOL format.

### 4.3. Quantitative Structure-Activity Relationship: OSIRIS

The cytotoxicity risk study was performed using OSIRIS DataWarrior 4.7.3 [138]. The cytotoxic effects were mutagenicity, carcinogenicity, and irritability to the skin and reproductive system. The TPSA (topological polar surface area) values were used to calculate the rate of absorption (%) of flavonoids and control as drugs by the formula

(1) $$\begin{array}{c} \%ABS=109-\left(0.345\times TPSA\right). \end{array}$$

### 4.4. Molecular Docking

For Parkinson's disease, the structures of human adenosine receptor A_2A_ (PDB ID 3UZA, at a resolution of 3.2 Å), *α*-synuclein (PDB ID 1XQ8), COMT (PDB ID 1H1D, at a resolution of 2 Å), and MAO-B (PDB ID 2C65, at a resolution of 1.7 Å) were downloaded from the Protein Data Bank (PDB) [139]. The choice of these proteins relied on protein validations reported in the literature, with anti-Parkinson activity as a prerequisite. The adenosine receptor A_2A_, COMT, and MAO-B proteins, respectively, contained 6-(2,6-dimethylpyridin-4-yl)-5-phenyl-1,2,4-triazin-3-amine (T4G) (an inhibitory drug), 1-(3,4,dihydroxy-5-nitrophenyl)-3-{4-[3-(trifluoromethyl) phenyl] piperazin-1-yl}propan-1-one (BIA), and ladostigil which served as bases for active site labeling and as control compounds for comparing energy values with the flavonoids. As for the *α*-synuclein protein, the option was chosen to detect 10 possible cavities, admitted as possible active sites on which to run the molecular docking. In order to compare the results of the 39 flavonoids, the docking was also run with the compound CLR01, an *α*-synuclein inhibitor from the literature.

For Alzheimer's, 4 targets with respect to pathology were analyzed, PDB ID 160 K (resolution of 1.94 Å) the crystal structure of glycogen synthase kinase 3 (GSK-3) with a complexed inhibitor [114], PDB ID 2FV5 (2.1 Å resolution) for the TACE crystal structure complexed with IK682 [140], PDB ID 3BKL (resolution 2.18 Å) for the ACE cocrystal structure with kAW inhibitor [141], and PDB ID 4DJU (resolution 1.8 Å) for the crystalline structure of BACE bound to 2-imino-3-methyl-5,5-diphenylimidazolidin-4-one [142]. The targets were selected based on scientific papers on *in silico* studies of molecules with anti-Alzheimer activity. The inhibitor for GSK-3 complexed together with the crystal structure was N-(4-methoxybenzyl)-N′-(5-nitro-1,3-thiazol-2-yl) urea (TMU), for TACE it was (2R)-N-hydroxy-2-[(3S)-3-methyl-3-{4-[(2-methylquinolin-4-yl)methoxy] phenyl}-2-oxopyrrolidin-1-yl] propanamide (541), and for ACE it was N-{(5S)-4,4-dihydroxy-6-phenyl-5-[(phenylcarbonyl)amino] hexanoyl}-L-tryptophan (kAW).

All 39 flavonoid structures (in MOL format) were submitted to molecular docking using the Molegro Virtual Docker v. 6.0.1 (MVD) [143]. All of the water compounds were deleted from the enzyme structure. For the molecular docking simulation, the bonds for all the compounds and the protein residues in the binding site were set as flexible, with a tolerance of 1.0, strength of 0.80, and with the torsional degrees of freedom for the flexible residues and ligands at 2000 steps of energy minimization. The enzyme and compound structures were prepared using the same default parameter settings in the same software package (score functions: MolDock score; ligand evaluation: internal ES, internal HBond, were all verified; number of runs: 10; algorithm: MolDock SE; maximum interactions: 1500; max. population size: 50; max. steps: 300; neighbor distance factor: 1.00; max. number of poses returned: 5). The docking procedure was performed using a 15 Å radius GRID and 0.30 of resolution to cover the ligand-binding site of the protein. For pose organizer, the MolDock score (GRID) algorithm was used as the score function and the Moldock search algorithm was used.

## 5. Results and Discussion

### 5.1. Quantitative Structure-Activity Relationship

Studies in structure-based design have become routine in drug discovery, searching for the best profiles against a disease. Thus, it is possible to analyze and discover various pharmacophoric groups and predict possible activities against a certain target. This study was performed through analysis of the physicochemical properties of drugs, such as TPSA and drug absorption, and using studies related to structure-based protein drug design. Toxicity risks and TPSA data, calculated in Osiris software, are presented in Table 2.

Mutagenicity studies can be used to quantify the role played by various organics in promoting or interfering with the way a drug can associate with DNA. According to the data from the Osiris program, flavonoids present low tendencies to be toxic. There were only six compounds that presented mutagenic toxicity (fisetin, genistein, gossypetin, hibiscetin, morin, and rhamnetin); two presented reproductive toxicity (genistein and procyanidin) and one presented tumor activity (genistein). These compounds present high risk and do not possess good drug profiles.

### 5.2. Molecular Docking in Parkinson's Disease

The molecular docking studies for the flavonoids and the control drugs with the PD targets are presented in Table 3.

For the enzyme Aden_2A_, it was observed that the three flavonoids (epicatechin gallate, hesperidin, and procyanidin) with respective energy values of −113.727 kcal/mol, −101.446 kcal/mol, and −98.216 kcal/mol presented higher affinities when compared to the PDB ligand (4TG).

**Figure 2:**
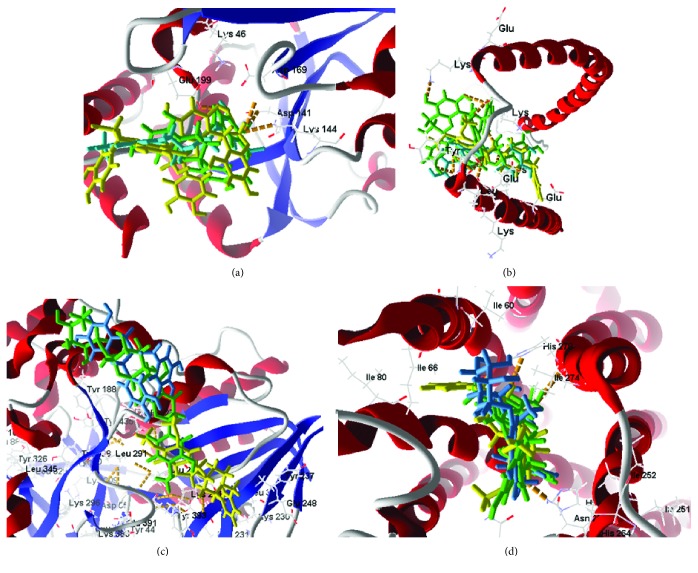
Molecular docking of flavonoids at the active site of Aden A_2A_ (PDB: 3UZA), *α*-synuclein (PDB: 1XQ8), COMT (PDB: 1H1D), and MAO-B (PDB: 2C65). (a) Docking of flavonoids at the active site of Aden_2A_ (green to epicatechin gallate, yellow to procyanidin, and blue to hesperidin). (b) Docking of flavonoids at the active site of *α*-synuclein (green to procyanidin, yellow to epicatechin gallate, and blue to rosinidin). (c) Docking of flavonoids at the COMT active site (green to epicatechin gallate and yellow to procyanidin and blue to europinidin) (ligand PDB). (d) Docking of flavonoids at the active site of MAO-B (green to hesperidin, yellow to epicatechin, and blue to aspalathin).

The flavonoids pre PDB ligand; hydrogen bonds present in hydroxyl groups with residues Asn253, Ala63, His250, His278, and steric interactions were observed for Asn253, Phe168, Trp246, and Leu249 for the flavonoids which presented higher score values. Key interactions were detected at His278, Leu249, and Asn253, being present in all of the flavonoids studied, principally at residue Asn253, because it is also present for the ligand PDB (Figure 2(a)).

For the enzyme *α*-synuclein, the observed value of PDB (CLR01 = −147.800 kcal/mol) presented better energy values as compared to flavonoids in the study. However, three of the compounds presented energy values close to that of the PDB ligand; these were procyanidin (−130.002 kcal/mol), epicatechin gallate (−98.330 kcal/mol), and rosinidin (−95.587 kcal/mol). For the flavonoids, hydrogen bonds were present for Lys43, Leu38, and Glu35. Key interactions were also observed for flavonoid activity in hydroxyl group steric interactions with residues Lys32, Lys43, and Glu35 considered key interactions for complex formation. These residues also appeared for the PDB ligand (Figure 2(b)).

Most COMT inhibitors have a catechol ring in their structure, such as entacapone and tolcapone, the most famous COMT inhibitor drugs. In our studies the enzyme COMT also presented flavonoid compound activity, being epicatechin gallate (−96.205 kcal/mol) a stronger interaction than the PDB ligand (BIA = −80.800 kcal/mol). For flavonoid activity, interactions with the active site presented eight residues, such as Asp141, Asn170, Lys144, Met40, and Glu199, forming hydrogen interactions with the catechol portions of the flavonoids. Residues Asn170, Glu199, Trp38, Leu198, Asp141, and Trp143 presented hydrophobic interactions with the hydroxyl portions of the flavonoids (Figure 2(c)). Similar results have been presented by Lee and Kim [144] and Tervo et al. [145], using molecular docking applied to compounds containing catechol and revealing potent COMT inhibition, which highlight the presence of these same interactions previously reported. The residues Asp141 and Asn170 were present for all of the flavonoids in our study, including the PDB ligand, making them key residues for the activity of these compounds.

MAO-B enzyme docking was performed at the two active PDB ligand sites. At the active site we saw that all of the flavonoids in the study were bound to the enzyme at both sites, with the same prevalence of compounds and presenting very close values at both sites. We also observed that the B subunit presents greater interaction with the compounds than subunit A (Table 3). Comparing the subunit B values, we found that the 10 flavonoid interactions were even more active than the PDB binder (4CR = −140 kcal/mol): 3-O-methylquercetin (−140.763 kcal/mol), 8-prenylnaringenin (−145.425 kcal/mol), aspalathin (−150.386 kcal/mol), capensinidin (−140.926 kcal/mol), europinidin (−140.585 kcal/mol), epicatechin gallate (−174.333 kcal/mol), hesperidin (−181.222 kcal/mol), homoeriodictyol (−141.639 kcal/mol), rosinidin (−149.196 kcal/mol), and sterubin (−141.623 kcal/mol). All these compounds presented steric interactions with residues Cys172, Tyr435, Leu171, Tyr435, Tyr326, Tyr60, and Gln206. Hydrogen bonds at Tyr398 and Met436 and Cys397 were presented with the hydroxyl portions of the flavonoids (Figure 2(d)). Similar results were also reported by Turkmenoglu et al. [39], using differing flavonoid derivatives in molecular docking (from a *Sideritis* species) for human monoamine oxidase (hMAO) isoform A and B, and by Shireen et al. [146] using flavonones from *Boesenbergia rotunda* for monoamine oxidase B, both of which presented interactions similar to our studied flavonoids, with docking results that presented significant hMAO-B inhibitory activity. Such activity is recommended for first-line drugs to treat Parkinson's disease.

We observed that the interactions between flavonoids and the study proteins occurred close to the hydroxyl groups present in the ligand structure and a strong interaction with the catechol ring. It was also observed that molecules with greater molecular mass, and electron-donating hydrophilic hydroxyl groups in ring position B, were more reactive with the enzyme, this, given the greater number of steric and electrostatic interactions with the catalytic site. The observations led to the hypothesis that such clusters can be viewed as possible pharmacophores for the development of anti-PD drugs.

Our screening results (yielding the best values against the four studied proteins) indicated that 8-prenylnaringenin, europinidin, epicatechin gallate, homoeriodictyol, capensinidin, and rosinidin present structural characteristics which guarantee their potential pharmacological activity against PD.

### 5.3. Molecular Docking in Alzheimer's Disease

Molecular docking of the 39 flavonoids was performed to analyze ligand-receptor integration for AD targets; the total interaction energy values are presented in Table 4.

For the GSK-3 target, two flavonoids (procyanidin and epicatechin gallate) presented better receptor interaction results with respective energy values of −115.164 kJ/mol and− 105.952 kJ/mol. However, procyanidin presents toxicity risks to the reproductive system. Analyzing interactions with the amino acid residues, we perceived hydrogen bonds of hydroxyls at residue Val135, as well as Asp133, and discretely at Arg141, Pro136, and Try134 for most of the studied flavonoids. Comparing the common amino acid residues of the interaction of the complexed ligand with the crystalline target, we noticed the common contribution of two residues with hydrogen bonds, 2 interactions with residue Val135, and 1 interaction with Pro136, leading to the hypothesis that these residues contribute to GSK-3 inhibitory activity.

For the TACE target, three flavonoids presented interaction energies below 150.0000 kJ/mol (epicatechin gallate, procyanidin, and aspalathin) with respective interaction energies of −187.352 kJ/mol, −154.184 kJ/mol, and− 153.001 kJ/mol. In addition to the abovementioned toxicity of procyanidin, there is little possibility for oral absorption since the %ABS = −5.241. For this target the molecules showed an interaction tendency for hydrogen bonding with Try433, Try436, and Pro437. For most of the compounds studied, the ligand when complexed with the PDB presented hydrogen-bonding interactions with residues Gly349, His409, His405, Glu406, Leu348, Gly349, and Asn447.

For the ACE target, thirteen compounds presented better interactions (below the median dock energy for each target studied) and hydrogen bond interactions with at least one of the amino acid residues: Tyr520, His513, Lys511, Tyr523, His353, Glu411, Glu384, and Ala356. Of these, five had molecular docking energies below −100.000 kJ/mol, aspalathin, epicatechin gallate, rosinidin, europinidin, and capensinidin.

**Figure 3:**
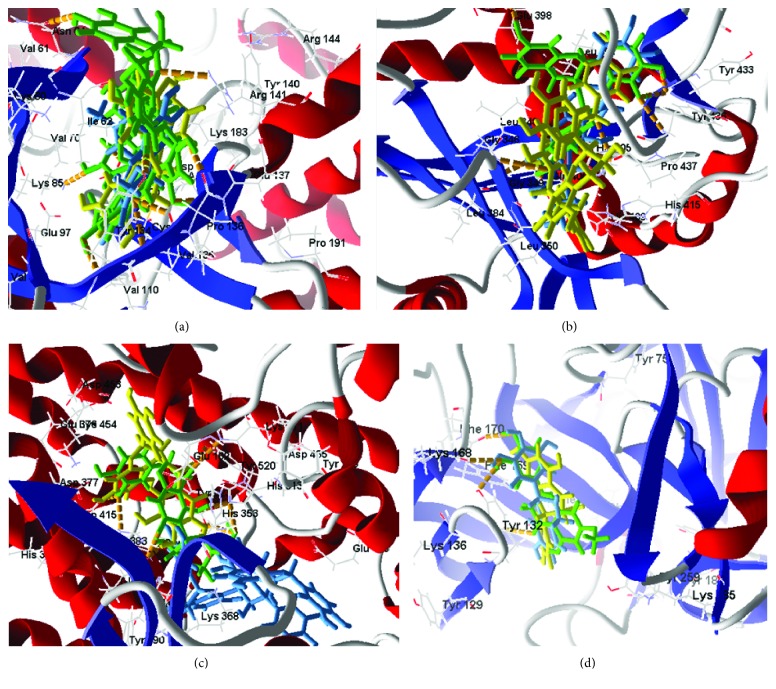
Molecular docking of flavonoids in the active site of GSK3 (PDB: 1Q5K), TACE (PDB: EFV5), ACE (PDB: 3BKL), and BACE1 (PDB: 4DJU). (a) Docking of flavonoids in the active site of GSK3 (green to procyanidin and yellow to epicatechin gallate). (b) Docking of flavonoids in the active site of TACE (green to epicatechin gallate, yellow to procyanidin, and blue to aspalathin). (c) Docking of flavonoids in the active site of ACE (green to aspalathin, yellow to epicatechin gallate, and blue to procyanidin). (d) Docking of flavonoids in the active site of BACE1 (green to sterubin, yellow to aromadendrin, and blue to robinetidinol).

Finally, for the BACE1 inhibition study, seventeen molecules presented satisfactory molecular docking energies, of which six (aromadendrin, sterubin, robinetidinol, capensidin, butin, and norartocarpetin) presented energies between −106.335 kJ/mol and −145.179 kJ/mol. The amino acid residues involved in the ligand-receptor interaction, with hydrogen bonds in important residues, Ile187, Glu95, Thr292, Asp289, Phe169, Thy132, Asn98, Trp137, Ser97, and Arg189, appeared with a high number of molecular bonds. In Figure 3, the docking of the 3 flavonoid enhancements for each target is presented.

By cross-checking the virtual screening data of the 39 flavonoids with the best interactions for each chosen PDB target, 7 flavonoids with the best results were obtained and are presented in this research: 8-prenylnaringenin, europinidin, epicatechin gallate, homoeriodictyol, aspalathin, butin, and norartocarpetin.

## 6. Conclusions

We conclude that the flavonoids of the study demonstrate potential neuroprotective activity by virtue of binding to certain key targets for Parkinson's and Alzheimer's. Based on our molecular docking studies, the flavonoids 8-prenylnaringenin, europinidin, epicatechin gallate, homoeriodictyol, capensinidin, and rosinidin present the best results for Parkinson's, whereas for Alzheimer's, the flavonoids 8-prenylnaringenin, europinidin, epicatechin gallate, homoeriodictyol, aspalathin, butin, and norartocarpetin present the best results. With lower and comparable binding energies (compared to crystallized binders), four flavonoids were observed in common for both diseases, presenting interactions and similarities consistent to those reported in the literature. For these flavonoid derivatives, it was observed that having greater flexibility together with hydrophobic hydroxyl groups facilitates interactions with hydrophobic regions of the target protein-binding sites.

## Figures and Tables

**Table 1:** Structure, name, structural formula, and molar mass of the flavonoids present in the study.

No.	Structure	Molecular name	Molecular formula	Mass
1	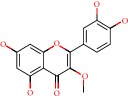	3-O-Methylquercetin	C16H12O7	316.058
2	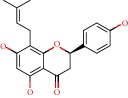	8-Prenylnaringenin	C20H20O5	340.131
3	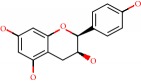	Afzelechin	C15H14O5	274.084
4	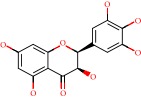	Ampelopsin	C15H12O8	320.053
5	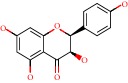	Aromadendrin	C15H12O6	288.063
6	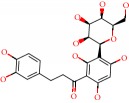	Aspalathin	C21H24O11	452.131
7	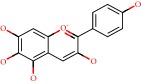	Aurantinidin	C15H11O6	287.055
8	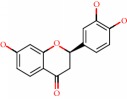	Butin	C15H12O5	272.068
9	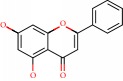	Capensinidin	C18H17O7	345.097
10	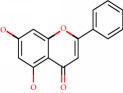	Chrysin	C15H10O4	254.057
11	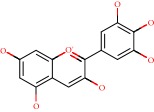	Delphinidin	C15H11O7	303.050
12	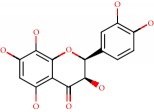	Di-hydrogossypetin	C15H12O8	320.053
13	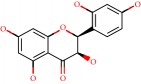	Di-hydromorin	C15H12O7	304.058
14	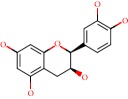	Epicatechin	C15H14O6	290.07
15	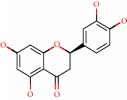	Eriodictyol	C15H12O6	288.063
16	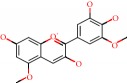	Europinidin	C17H15O7	331.081
17	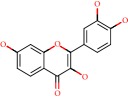	Fisetin	C15H10O6	286.047
18	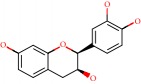	Fisetinidol	C15H14O5	274.084
19	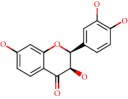	Fustin	C15H12O6	288.063
20	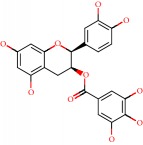	Epicatechin gallate	C22H18O10	442.090
21	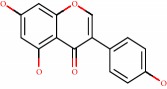	Genistein	C15H10O5	270.052
22	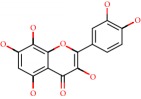	Gossypetin	C15H10O8	318.037
23	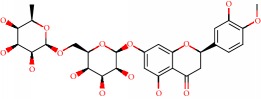	Hesperidin	C28H34O15	610.189
24	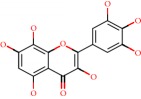	Hibiscetin	C15H10O9	334.032
25	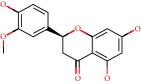	Homoeriodictyol	C16H14O6	302.079
26	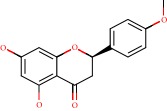	Isosakuranetin	C16H14O5	286.084
27	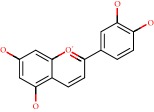	Luteolinidin	C15H11O5	271.060
28	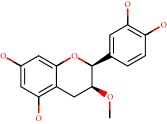	Meciadanol	C16H16O6	304.094
29	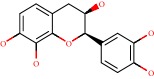	Mesquitol	C15H14O6	290.079
30	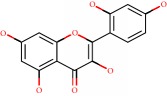	Morin	C15H10O7	302.042
31	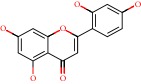	Norartocarpetin	C15H10O6	286.047
32	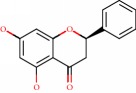	Pinocembrin	C15H12O4	256.073
33	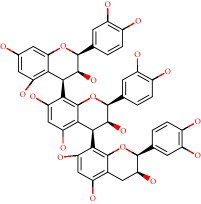	Procyanidins	C45H38O18	866.205
34	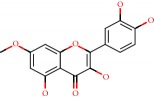	Rhamnetin	C16H12O7	316.058
35	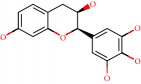	Robinetidinol	C15H14O6	290.079
36	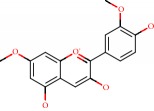	Rosinidin	C17H15O6	315.086
37	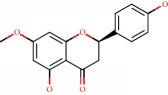	Sakuranetin	C16H14O5	286.084
38	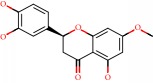	Sterubin	C16H14O6	302.079
39	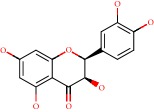	Taxifolin	C15H12O7	304.058
40	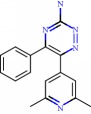	Control 4TG-Aden2A-Parkinson	C17H27N3O15P2	575.357
41	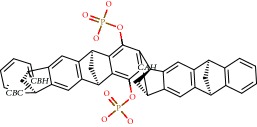	Control CLR01–Parkinson	C42H32O8P2	726.658
42	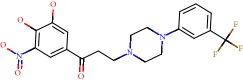	Control BIA–Parkinson	C16H20N4O2	300.360
43	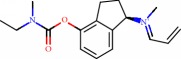	Control ladostigil-Parkinson	C16H20N2O2	272.340
44	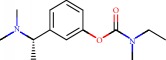	Control rivastigmine-Alzheimer	C14H22N2O2	250.337
45	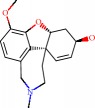	Control galantamine-Alzheimer	C17H21NO3	287.340
46	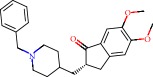	Control donepezil-Alzheimer	C24H29NO3	379.480

**Table 2:** Toxicity data, TPSA, and %ABS calculated on the Osiris tool for flavonoids.

Flavonoids	Toxicity risks	TPSA	%ABS
3-O-methylquercetin	No	116.450	68.824
8-prenylnaringenin	No	86.990	78.9884
Afzelechin	No	90.150	77.8982
Ampelopsin	No	147.680	58.050
Aromadendrin	No	107.220	72.009
Aspalathin	No	208.370	37.112
Aurantinidin	No	101.150	74.103
Butin	No	86.990	78.988
Capensinidin	No	88.380	78.508
Chrysin	No	66.760	85.967
Delphinidin	No	121.380	67.123
Di-hydrogossypetin	No	147.680	58.050
Di-hydromorin	No	127.450	65.029
Epicatechin	No	110.380	70.918
Eriodictyol	No	107.220	72.009
Europinidin	No	99.380	74.713
Fisetin	Mutagenic	107.220	72.009
Fisetinidol	No	90.150	77.898
Fustin	No	107.220	72.009
Epicatechin gallate	No	177.140	47.886
Genistein	Mutagenic/tumor/reproductive	86.990	78.988
Gossypetin	Mutagenic	147.680	58.050
Hesperidin	No	234.290	28.169
Hibiscetin	Mutagenic	167.910	51.071
Homoeriodictyol	No	96.220	75.804
Isosakuranetin	No	75.990	82.783
Luteolinidin	No	80.920	81.082
Meciadanol	No	99.380	74.713
Mesquitol	No	110.380	70.918
Morin	Mutagenic	127.450	65.029
Norartocarpetin	No	107.220	72.009
Pinocembrin	No	66.760	85.967
Procyanidin	Reproductive	331.140	−5.243
Rhamnetin	Mutagenic	116.450	68.824
Robinetinidol	No	110.380	70.918
Rosinidin	No	92.290	77.159
Sakuranetin	No	75.990	82.783
Sterubin	No	96.220	75.804
Taxifolin	No	127.450	65.029

**Table 3:** Description of energy scores of flavonoids and control compounds on PD target proteins.

Flavonoids	Aden A_2A_	*α*-Synuclein	COMT	MAO-B
3-O-methylquercetin	−71.095	−74.901	−53.659	−140.763
8-prenylnaringenin	−83.692	−83.012	−67.998	−145.425
Afzelechin	−61.973	−70.911	−51.278	−107.22
Ampelopsin	−60.848	−74.188	−53.806	−134.626
Aromadendrin	−53.880	−66.701	−45.951	−123.726
Aspalathin	−55.009	−86.361	−56.396	−150.386
Aurantinidin	−67.749	−75.414	−56.591	−117.977
Butin	−68.355	−77.949	−60.034	−124.25
Capensinidin	−84.669	−87.321	−71.529	−140.926
Chrysin	−59.594	−70.872	−52.576	−120.287
Delphinidin	−70.457	−82.877	−68.376	−126.481
Di-hydrogossypetin	−56.359	−73.612	−48.949	−135.483
Di-hydromorin	−61.416	−66.071	−54.329	−131.088
Epicatechin	−66.996	−74.661	−53.054	−122.78
Eriodictyol	−66.790	−74.167	−55.545	−119.801
Europinidin	−75.421	−79.694	−74.993	−140.585
Fisetin	−67.182	−79.763	−64.252	−130.773
Fisetinidol	−64.279	−72.271	−59.406	−118.506
Fustin	−59.854	−76.510	−56.851	−135.63
Epicatechin gallate	−113.727	−98.330	−96.205	−174.333
Genistein	−68.316	−73.585	−58.867	−119.162
Gossypetin	−63.019	−75.620	−58.446	−139.059
Hesperidin	−101.446	−89.698	−65.656	−181.222
Hibiscetin	−71.879	−75.302	−60.718	−137.019
Homoeriodictyol	−75.599	−82.786	−62.698	−141.639
Isosakuranetin	−65.924	−71.351	−49.177	−131.514
Luteolinidin	−65.240	−80.031	−57.149	−122.481
Meciadanol	−73.596	−77.668	−55.342	−126.337
Mesquitol	−60.219	−74.776	−51.753	−128.058
Morin	−70.744	−84.587	−59.595	−139.778
Norartocarpetin	−67.527	−77.898	−60.514	−137.774
Pinocembrin	−56.707	−66.573	−46.254	−113.423
Procyanidin	−98.216	−130.002	−85.226	−88.460
Rhamnetin	−69.702	−83.582	−49.586	−142.785
Robinetinidol	−62.594	−78.967	−51.172	−125.203
Rosinidin	−83.735	−95.587	−63.376	−149.196
Sakuranetin	−70.695	−74.984	−51.408	−129.56
Sterubin	−69.560	−77.022	−56.015	−141.623
Taxifolin	−56.665	−69.743	−52.804	−126.612

**Table 4:** Energy scores of flavonoids and control compounds against Alzheimer's disease.

Name	1Q5K	2FV5	3BKL	4DJU
3-O-Methylquercetin	−77.844	−137.815	−89.583	−81.959
8-Prenylnaringenin	−97.365	−132.520	−96.493	−85.052
Afzelechin	−69.480	−120.893	−79.982	−65.259
Ampelopsin	−71.079	−119.645	−83.823	−68.341
Aromadendrin	−65.678	−115.123	−81.374	−145.179
Aspalathin	−91.374	−153.001	−125.583	−92.594
Aurantinidin	−77.482	−113.425	−84.517	−60.915
Butin	−80.350	−132.235	−89.736	−110.684
Capensinidin	−77.262	−134.112	−108.407	−118.415
Chrysin	−77.346	−117.834	−88.051	−85.052
Delphinidin	−86.937	−132.828	−98.687	−73.381
Di-hydrogossypetin	−66.795	−120.679	−80.429	−72.832
Di-hydromorin	−67.026	−121.489	−87.870	−71.631
Donepezil^∗^	−112.609	−154.722	−119.399	−83.404
Epicatechin	−72.393	−127.619	−83.552	−78.328
Eriodictyol	−74.681	−124.042	−87.631	−90.944
Europinidin	−85.511	−140.803	−108.977	−89.075
Fisetin	−81.627	−139.645	−95.587	−73.317
Fisetinidol	−74.131	−116.368	−83.084	−65.914
Fustin	−74.571	−116.130	−80.078	−74.650
Galantamine^∗^	−84.430	−156.068	−93.838	−115.428
Epicatechin gallate	−105.952	−187.352	−114.841	−83.154
Genistin	−78.990	−127.356	−89.509	−90.625
Gossypetin	−69.944	−142.715	−84.131	−79.410
Hesperidin	−85.551	−145.093	−97.557	−80.780
Hibiscetin	−66.573	−144.530	−103.117	−81.446
Homoeriodictyol	−85.345	−134.677	−93.198	−82.368
Isosakuranetin	−76.492	−124.546	−81.779	−70.443
Luteolinidin	−76.499	−121.014	−84.251	−87.799
Meciadanol	−73.882	−127.300	−84.290	−74.119
Mesquitol	−81.114	−130.982	−92.321	−80.051
Morin	−79.444	−130.332	−97.326	−80.051
Norartocarpetin	−79.739	−128.750	−99.216	−106.335
Pinocembrin	−67.298	−113.647	−81.535	−56.405
Procyanidin	−115.164	−154.184	−113.990	−81.313
Rhamnetin	−81.950	−127.432	−89.885	130.736
Rivastigmine^∗^	−76.582	−121.774	−85.559	186.829
Robinetidinol	−86.339	−124.910	−95.178	−136.143
Rosinidin	−96.375	−134.734	−111.602	266.611
Sakuranetin	−74.645	−118.156	−88.698	−89.075
Sterubin	−85.628	−124.397	−91.209	−145.179
Taxifolin	−69.263	−120.177	−77.806	−82.368

^∗^Drugs used as a control for Alzheimer's molecular docking.

## Data Availability

The data used to support the findings of this study are available from the corresponding author upon request.
